# Unmet Needs and Coping Mechanisms Among Community-Dwelling Senior Citizens in the Philippines: A Qualitative Study

**DOI:** 10.3390/ijerph16193745

**Published:** 2019-10-04

**Authors:** Rogie Royce Carandang, Edward Asis, Akira Shibanuma, Junko Kiriya, Hiroshi Murayama, Masamine Jimba

**Affiliations:** 1 Department of Community and Global Health, Graduate School of Medicine, The University of Tokyo, Tokyo 113-0033, Japan; rrcarandang@gmail.com (R.R.C.); shibanuma@gmail.com (A.S.); jkiriya@m.u-tokyo.ac.jp (J.K.); 2 Institute of Gerontology, The University of Tokyo, Tokyo 113-8656, Japan; murayama@iog.u-tokyo.ac.jp; 3 Department of Global Studies, Faculty of Liberal Arts, Sophia University, Tokyo 102-8554, Japan; edward.asis2015@gmail.com

**Keywords:** unmet needs, coping, senior citizens, aging, qualitative, Philippines

## Abstract

The Philippine government is facing a burden of improving health and social services for both the growing elderly and young population. The extent of discussion on aging issues and concerns, however, is minimal at best. Therefore, this study aimed to examine the perceptions of unmet needs and to explore the coping mechanisms of senior citizens across local stakeholders in an urban area in the Philippines. In this qualitative study, we collected data using focus group discussions among senior citizens (*n* = 4) and health providers (*n* = 4) as well as in-depth interviews among local administrators (*n* = 7). We analyzed the data through thematic analysis using the social determinants of health as the theoretical framework. We used qualitative research software NVivo10® to track the coding and manage the data. Four major themes related to unmet needs emerged in the analyses: (1) financial security, (2) health care services, (3) age-friendly environment, and (4) family support. Senior citizens responded either positively or negatively to cope with the challenges associated with aging. The government must then revisit existing national policies to address their unmet needs. Actions should be taken to strengthen positive coping and modifying the negative coping mechanisms, with a particular focus on community and family interventions.

## 1. Introduction

The Asia-Pacific region is experiencing profound and rapid demographic changes [1]. The aging process is changing at an unprecedented rate, and the timing and pace of this transition vary across the region. The Philippines, for instance, has a simultaneously growing elderly and young population. The proportion of persons aged 60 years and above will increase from 6.7% of the population in 2010 to 11.4% in 2030 [2,3]. The population aged 0–14 years will constitute 26.8% of the population by 2030 [2]. Therefore, this demographic change can put a strain on the country’s resources as the government is facing a burden of improving health and social services. It is then a matter of prioritization which segment of the population will be focused on by the government.

Little has been investigated on Filipino senior citizens. To date, only two nationally representative surveys were conducted in 1996 and 2007, which highlighted their health status and well-being [4,5]. Carlos [6] noted that research on senior citizen issues, such as their social security and poverty, health concerns, and abuse, is relatively limited. She said it might be because they are a small minority of the population as compared to the youth. Moreover, De Leon [7] stated that the extent of the issues and concerns of the aging population had been mainstreamed in discussions on Philippine development is minimal at best. She added that although the government has initiated social welfare actions, these continue to face challenges in its implementation, primarily because of the low appreciation of their issues [7].

At present, the heavier weight of responsibility of caring for the senior citizens is on the shoulders of Filipino families, not on the government [7]. This delegation is reflected in the Philippine constitution [8], which states that "*The family has the duty to care for its elderly members, but the State may also do so through just programs of social security”* (Article XV, Section 4). However, issues, such as the rising cost of basic commodities, education, healthcare, and recreation, among others, directly or indirectly affect the welfare of Filipino senior citizens. Their needs are often foregone by the household in favor of its younger or employed members [7,9].

Two major laws were enacted to address senior citizen concerns, namely, RA 7876 and RA 9994. RA 7876 mandates the establishment of the Office of the Senior Citizens Affairs (OSCA) in all Local Government Units (LGUs), while RA 9994 provides for a 20% discount on basic food and medicine purchases [10,11]. Under the office of the mayor, the OSCA has the primary task of facilitating the release of senior ID’s, which are required for availing discounts and provides avenues for the senior citizens to engage in social and recreational activities. Moreover, the law does not preclude LGUs from broadening the scope of subsidized services and goods for the senior citizens in consideration of the specific needs and issues of the population in their area [7]. Therefore, it is essential to explore the actual needs and preferences of senior citizens to identify which assistance and services to provide that will improve their quality of life.

Unmet needs are defined as the difference between services deemed necessary by the recipients and the actual services they receive and represent a measure of the quality of life [12,13]. Several studies focused on the unmet needs of senior citizens diagnosed with cancer and undergoing active cancer treatment [14]. For example, according to a study conducted by Hansen et al. [15], having unmet needs of rehabilitation was strongly associated with more psychological distress and reduced health-related quality of life. Only a few studies, however, focused on community-dwelling senior citizens. A study in Spain stated that there were considerable social and material inequalities in access to home care [16]. These inequalities may widen in the future due to poor coverage of the public system for home and personal care [16]. Thus, the lack of public policy initiatives may lead to increasing unmet needs. Generally, senior citizens have more complex needs compared with younger adults due to an additional disability, physical illness, and social needs [12]. Their unmet needs should be identified and anticipated due to its economic and social consequences. By understanding the nature and extent of their unmet needs, the government can formulate new public policy initiatives.

Little is known about how Filipino senior citizens cope with their unmet needs. Coping mechanisms are the strategies people often use in the face of adversities to help manage painful and challenging life events [17] while maintaining their emotional and psychological well-being [18]. The recent literature suggested that coping may be a developmental and multifaceted process, and that specific coping mechanisms used by senior citizens is not as clear-cut [19]. Positive coping mechanisms (e.g., information seeking, taking action) may have positive and even protective effects on health, whereas negative coping mechanisms (e.g., distraction, venting) have adverse effects [19]. By exploring Filipino senior citizens’ coping mechanisms, we can formulate strategies to promote resilience and help them address their unmet needs more effectively.

In this study, we aimed to examine the perceptions of unmet needs and to explore the coping mechanisms of senior citizens across three key local stakeholder groups (senior citizens, health providers, and local administrators) in an urban area in the Philippines.

## 2. Materials and Methods

### 2.1. Study Design

The qualitative study design was drawn on the interpretive paradigm, which explored the unmet needs and coping mechanisms of community-dwelling senior citizens in the Philippines. In this study, we used the social determinants of health as our theoretical framework to see the different conditions that help senior citizens maintain their health and well-being [20]. We had five dimensions of focus, which included economic aspects, health and social services, community factors, physical and social environments, and household issues. These were used for instrument development for each stakeholder group.

### 2.2. Study Participants

In this study, we focused on the data collection of three stakeholder groups (Table 1). The first group comprised of community-dwelling senior citizens. We conducted four focus group discussions (FGDs) with 7–10 members each to elicit their perceptions of unmet needs and coping mechanisms. The second group comprised of health providers in the barangays (it refers to communities in the Philippines) that include primary care physicians, nurses, midwives, and barangay health workers. We conducted four FGDs with 3–5 members each to understand health systems challenges and elicit their perceptions on the health needs of the senior citizens. The FGD method was chosen for both senior citizens and health providers because it could stimulate the group dynamics of the participants by providing the facilitator with rich data [21]. This context would not have been possible with one-to-one interviews, especially when there is little background on the participants [22]. The third group comprised of local administrators and included the city mayor, city council staff, barangay captains, and OSCA representatives. Due to their busy schedule, we conducted brief structured in-depth interviews with each of them (*n* = 7) instead of FGDs. We assessed their perspectives on health needs and community engagement of senior citizens, as well as challenges in the implementation of health and social services.

### 2.3. Data Collection

We approached the barangay captains to hand over formal invitations for FGDs. We were then referred to the Barangay Senior Neighborhood Organization (BSNO) and Barangay Health Center for recruitment. We organized a meeting with them to discuss the inclusion criteria for potential participants. The BSNO staff recruited senior citizens who were 60 years old and above and registered members of the OSCA. Senior citizens who were interested visited the barangay hall on the day of the FGDs. As for health providers, the primary care physician did the recruitment, and we had our FGDs at the Barangay Health Center. As for local administrators, we handed over appointment request letter and had in-depth interviews in their respective offices.

We based the data collection instruments (topic guides) for FGDs and in-depth interviews on the study objectives and theoretical framework. The topic guides ensured the inclusion of previously identified essential issues [23]. Probes were added to get further information on key aspects. Table 1 shows the areas of inquiry for the topic guide that included current economic and social security schemes, issues related to access to health services, community profile, built environment and social participation, and family relationships. Sample questions include: (1) what do you think about your health status? (2) what can you say about your community? (3) how is your relationship with your family members? The topic guides in both English and Filipino languages are available as Supplementary Files (Tables S1 and S2). FGDs lasted for about 60–90 min, whereas in-depth interviews lasted approximately an hour. Two investigators (R.R.C. and E.A.) conducted the FGDs and in-depth interviews with the assistance of a senior volunteer. We ceased conducting interviews when data saturation was reached, and that was when all transcripts from each stakeholder group had been coded, with no new codes emerging [24]. We collected the data from August to September 2017.

### 2.4. Data Analysis

All interviews were audio-recorded and transcribed verbatim in Filipino. Two of the authors (R.R.C. and E.A.) read the transcripts thoroughly and interpreted the data as a whole. The transcripts were subsequently analyzed using a thematic approach [25,26]. Qualitative research software NVivo10® (QSR International, Burlington, MA, USA)was used to track the coding and manage the data. We analyzed the transcripts in the following manner. First, we assigned codes to phrases and sentences that described the meaning of the text segment. Then, we assigned texts that had a similar meaning under the same code; otherwise, we gave them a new code. Based on our framework, the whole transcription was re-coded, and then we continued the process until no new code was extracted from the transcript. Next, we gathered similar codes into more conceptual categories. Finally, we identified themes by relating categories and subcategories. We validated the coding and themes through continuous dialogue among co-researchers and the peer debriefing to interpret results with geriatric healthcare professionals [27]. We provided feedback to the interviewees by giving a summary of the findings before finalizing the main themes and categories. They confirmed our results and provided valuable comments, helping us to refine our findings further. As for coping, senior citizens helped in categorizing them into either positive or negative mechanisms. After reaching consensus, we translated the identified themes and quotations to support themes into English.

The qualitative methods and reporting of results adhered to the Consolidated Criteria for Reporting Qualitative Studies (COREQ) guidelines [28] and Standards for Reporting Qualitative Research (SRQR) [29]. A complete COREQ checklist was also included (Table S3).

### 2.5. Ethics Statement

The study was approved by The University of Tokyo Research Ethics Committee (SN 11641) and by the University of the Philippines-Manila Research Ethics Board (UPMREB 2017-312-01). We explained the goal of the research to the interviewees, and their anonymity was guaranteed. All participation was voluntary, and we secured written informed consent before the interviews. We obtained all required permits and approvals as applied to foreign researchers.

## 3. Results

### 3.1. General Characteristics of Participants

We described the participants’ characteristics in Table 1. The four FGDs among the community comprised 7–10 senior citizens, with one-third of them as men. The four FGDs among health providers comprised 3–5 members, and all of them were women. The senior citizens’ age ranged from 60–85 years, whereas health providers’ age ranged from 38–69 years. In the in-depth interviews with local administrators, they were generally women (only one administrator was a man) with an age range of 38–65 years.

We presented the findings in two sections (Table 2 and Table 3), corresponding to the two aims of our study. We based the first section (unmet needs) on data from the FGDs with senior citizens and health providers, as well as in-depth interviews with local administrators. The results in section two (coping mechanisms) are based solely on the FGDs with senior citizens. Throughout the results, quotes are presented to illustrate the findings.

### 3.2. What Are the Identified Unmet Needs Among Filipino Senior Citizens?

Table 2 shows the analytical framework of perception of unmet needs of community-dwelling senior citizens. It includes the unmet need for financial security, health care services, age-friendly environment, and family support.

#### 3.2.1. Unmet Need for Financial Security

Financial security is a critical issue for senior citizens. Many of them mentioned that they were unable to save for their pension due to the high rates of the informal workforce in the Philippines. They also identified low household income as a challenge affecting their families. They stated that they have to prioritize their families’ survival over pension.

“*I was never employed in any formal and regular jobs when I was young; that is why I was not able to save for my pension. I did not have enough disposable income to save and prepare for my retirement*.”.

(senior citizen, 65 years old)

“*We had to budget our small income carefully. We had to prioritize food and other household needs over pension. We have to pay for electric bills and children’s education. Family first before ourselves*.”.

(senior citizen, 68 years old)

Meanwhile, the government has a social pension program, but it is only limited to indigent senior citizens. All of the senior citizens argued that the definition of the indigent is very subjective and that nepotism may appear in the selection of eligible recipients. Local administrators, however, clarified that nepotism does not exist in the implementation of the program. They emphasized fairness in their selection because they do home visits to validate the actual living conditions of the prospective beneficiaries.

“*Indigent means frail, sickly, no source of income, or no support from families or relatives. The local administrators have different interpretations, so there is palakasan (nepotism) in the implementation. If you know someone in the government, then your chances of getting [social pension] are higher. This palakasan will make equally poor and vulnerable senior citizens be left out*.”.

(senior citizen, 64 years old)

“*We prioritize senior citizens who met the eligibility criteria. There is no special treatment in our selection. We do home visits to validate whether the supposed recipient is indeed eligible to the program. The slots are limited, so we need to prioritize and choose the poorest of the poor*.”.

(local administrator, 65 years old)

The social pension is not universal and has a low benefit level. The government provides indigent senior citizens a monthly pension of 500 pesos (10 USD), which is paid as 1500 pesos every quarter. All of them suggested that all senior citizens must be entitled regardless of their socioeconomic status. They hoped for the implementation of a universal social pension. They added that although it is not enough, it helps them allocate budget for their basic needs (especially food and medicines). It also provides them a basic level of income security.

“*We will not live longer, so we want to enjoy the full and equal benefits before we die. As long as we turned 60, whether we are rich or poor, we must be eligible for the social pension. We hope it to be universal. This is one way for the government to show their appreciation to us*.”.

(senior citizen, 60 years old)

“*The social pension is not enough to buy for my food and maintenance drugs, but 1500 pesos (30 USD) quarterly can make a difference. It gives me a sense of security because I know I have something to look forward to. It is better than nothing, so I am thankful to be one of the beneficiaries. However, if the social pension is bigger, I would be able to buy more and help in my family’s expenses*.”.

(senior citizen, 72 years old)

As for those senior citizens who were not receiving any pension, they had to continue working until they could. Job opportunities, however, are limited for this particular age group. Self-employment was common among them. The local administrators acknowledged the importance of future collaboration with the private sectors to create jobs for them.

“*I would like to continue working as long as I am physically able. I need to earn money not only for myself but also for my family. I cannot rely on any of my children because they are also poor like me*.”.

(senior citizen, 60 years old)

“*I acknowledge the lack of job opportunities for senior citizens. We need to form a partnership with the private sectors to come up with career opportunities appropriate for this age group*.”.

(local administrator, 62 years old)

#### 3.2.2. Unmet Need for Health Care Services

Health care services are said to be inadequate due to the following reasons: staffing problem, drug supply problem, and accessibility of the health center.

As for the staffing problem, senior citizens criticized the availability of doctors. They said that some barangays are sharing a doctor, so there were a few times that a doctor was absent in one barangay. Health providers explained the reason and said that new doctors are required to attend seminars for a couple of days, which is why they were absent.

“*We have no permanent doctor. Normally, health centers are sharing doctors. There were times that I went to the center, but the doctor was not around*.”.

(senior citizen, 70 years old)

“*New doctors attend seminars for a couple of days. We cannot find a substitute doctor, so midwives normally attend to the patients’ concerns. We do not let the patients go home without receiving care. We address their concerns as much as we can*.”.

(health provider, 40 years old)

As for the drug supply problem, most of the senior citizens said that the health center quickly runs out of supplies because of the high number of patients received every day. Partial filling of prescriptions has become the norm to compensate for the lack of supplies.

“*There are always too many patients in the health center. They have a limited supply of free medicines. Normally, they partially filled my prescription for my maintenance and asked me to buy the rest at a nearby pharmacy. However, I do not have the money, so I have to wait until the next supply*.”.

(senior citizen, 75 years old)

Health providers admitted that they do partial filling because there is a delay in the procurement of medicines from the LGU. However, once available, they freely dispense the medicines to all patients in need.

“*Yes, we partially filled their prescriptions. We sometimes experience a delay in the procurement, so we explain to the seniors the situation. We also informed them that they could receive a 20% discount on their prescription if they go to the nearby pharmacy. All they have to do is to show their senior ID and bring their booklet*.”.

(health provider, 44 years old)

Despite these efforts from the health providers, one senior citizen complained about the accessibility of the health center. She stated her need for home-based care services because of her frail condition and hesitation to bother her family members.

“*The [health] center is far from my place. I need someone to bring me there. I cannot go there by myself because I am already weak. I do not want to bother my family so they (health providers) should bring health care to our homes*.”.

(senior citizen, 80 years old)

#### 3.2.3. Unmet Need for an Age-Friendly Environment

Regarding the physical environment, all senior citizens emphasized the importance of a safe environment and good housing conditions. They perceived that a barrier-free environment is a vital aspect of their mobility and quality of life. One senior citizen talked about his struggle during the rainy season. He shared about building some wooden bridges so people can pass over the flood. Another senior citizen who lives in the squatter area talked about her poor housing condition. The local administrators said that it was a struggle to go to those places during outreach programs or gift-giving events. They said that the quality of life there is unfortunate and miserable.

“*Our area often gets flooded after heavy rain. We built wooden bridges so we can pass over the flood. I always cross the bridge and sometimes fall because it is slippery. I had minor fractures since then, but I got used to it eventually (laughs)*.”.

(senior citizen, 70 years old)

“*I sleep in a tiny room with poor ventilation. I lie on a mat together with my grandchildren. I always have back pain when I wake up. I wish we could live in a better house where I can sleep and move comfortably*.”.

(senior citizen, 60 years old)

“*We struggled to do home visits to the seniors living in squatter areas. Their housing condition is poor, and seniors do not live comfortably there as they do not have sufficient space and privacy. The quality of life there is unfortunate and poor. I wish we could do more for them*.”.

(local administrator, 44 years old)

Concerning the social environment, senior citizens would like the OSCA to be more active in providing geriatric services. They suggested the inclusion of community-based programs and recreational activities to be available all year round. OSCA representatives, however, mentioned that the idea was great, but the budgetary constraint is the biggest hurdle for program implementation. Moreover, there is only one OSCA per city or municipality in the Philippines. This situation is another reason why there are few opportunities for senior citizens to join social events. Senior citizens suggested building a senior center for each barangay.

“*Occasional field trips and seminars could only reach a few interested individuals. If the OSCA would be more active in implementing various daily or weekly activities, then more seniors can participate*.”.

(senior citizen, 63 years old)

“*Having various weekly activities are great, but it is the LGU which approves our budget. We propose and implement programs that fit our approved budget. If we had a bigger budget, we could launch more projects*.”.

(local administrator, 40 years old)

“*Each barangay must have one senior hall so we can gather and hold some meetings or events anytime we wish. This hall will serve as a venue for gatherings or socialization*.”.

(senior citizen, 66 years old)

#### 3.2.4. Unmet Need for Family Support

The reduction of family support is brought by the Overseas Filipino Worker (OFW) phenomenon, parenting by proxy, children’s lifelong dependency, and limited family bonding time.

Some senior citizens talked about the direct implication of the OFW phenomenon in geriatric care. Some of them mentioned that children’s migration abroad often leads to neglect of their senior parents. Health providers agreed to that and further commented about the consequences of migration of traditional caregivers and the growing influx of nurses and other health professionals to other countries with an advanced aging population.

“*There is no provision of physical care when your children live abroad. It is difficult to find surrogate family caregivers who are willing to take on the daily task of elderly care. So, my wife and I rely on each other*.”.

(senior citizen, 65 years old)

“*Daughters are known to be the traditional caregivers in the Philippines. However, due to overseas migration, seniors can no longer rely on them and thus, might lead to neglect. Our nurses and other health professionals were also moving abroad in search of a better future. Who will then take care of the frail and sick seniors? Caregiving has not been institutionalized in our country yet. It is in our culture that family members have to take care of the seniors*.”.

(health provider, 43 years old)

A few senior citizens talked about ‘parenting by proxy,’ which is a new trend in the Philippines as both husband and wife assume full-time jobs. One senior citizen shared about the stress associated with parenting her grandchildren. Another complained about her living arrangement and the lifelong dependence of her married children on her. She said that her parental obligations to support her adult children are still there.

“*My children left all their kids with me, and they are not giving any financial support. My husband and I are the ones raising them. It stresses me out because I do not know where to get money for my grandchildren’s education*.”.

(senior citizen, 62 years old)

“*All my children have families of their own, but we are all living under the same roof. They are even asking for financial help because they are poor. I thought I have already fulfilled my moral duty as a parent. I was wrong*.”.

(senior citizen, 65 years old)

A few senior citizens also mentioned about their limited family bonding time. They said that the reduction of face-to-face interaction among family members is due to greater dispersion of their children, both domestic and overseas. Advances in communication technology (e.g., mobile phone, social media) have made contact possible, but the frequency of contact is still insufficient. Another senior citizen remarked that even if their children live with them, their family bonding time is quite limited.

“*I am longing for my children. They live far away. I wish we could be complete again and be reunited, just like the old times*.”.

(senior citizen, 60 years old)

“*We do Facebook or skype, but we rarely communicate because they live abroad. The time difference is also a factor. When we talk, they are already tired and wanted to rest, so we keep the conversation short*.”.

(senior citizen, 61 years old)

“*My children are all busy at work. We rarely hang out together. The only time we catch up is when we are all sitting around the dining table. I hope we can sit and talk more about their personal life*.”.

(senior citizen, 64 years old)

### 3.3. What Are the Identified Coping Mechanisms Among Filipino Senior Citizens?

Table 3 shows the analytical framework of coping mechanisms practiced by community-dwelling senior citizens. Two types of coping mechanisms emerged in the analyses: (1) positive coping, such as self-reliance and religious involvement and (2) negative coping, such as self-medication, avoidance, homeboundness, longsuffering attitude, and overdependence.

#### 3.3.1. Positive Coping Mechanisms

##### Self-Reliance

Some senior citizens mentioned that the absence of pension and lack of financial assistance from their children pushed them to self-employment in later life. They mentioned that being self-reliant boosts their self-esteem and gives them financial security.

“*I do not have a pension, so I do direct selling, like buy and sell. I can earn a small amount of money to meet my basic needs, especially food and medicines. I depended on my abilities instead of relying on my children*.”.

(senior citizen, 68 years old)

“*I am not receiving any financial help from my children, so I work for myself. I am involved with rag making, dressmaking, and running my sari-sari store (neighborhood sundry store). Being independent enhances my self-image, and I can gain respect from my own family and community*.”.

(senior citizen, 67 years old)

“*I am involved in community civic engagement and getting a small monthly allowance in return. It gives me a sense of financial security*.”.

(senior citizen, 65 years old)

##### Religious Involvement

Senior citizens who were frequent churchgoers reported more extensive social networks and more types of social support received than their less religious counterparts. Some of them were active in doing bible study as well as singing in a chorus.

“*I am an active member of the church. I have a strong network of church friends with whom I can share my problems. Joining bible study with them and singing in a chorus bring joy and add meaning to my life*.”.

(senior citizen, 61 years old)

“*I do not often go to church. However, I guess there are a few people around me whom I can rely on*.”.

(senior citizen, 62 years old)

#### 3.3.2. Negative Coping Mechanisms

##### Self-Medication

Some senior citizens self-medicate due to lack of budget and supply problems at the health center. They used herbal supplements and decoctions to manage their illness and meet their treatment demands. They knew that self-medication is wrong but claimed that home remedies are more natural and cheaper than modern pharmaceuticals.

“*I boil some herbal plants because I cannot afford my maintenance [drugs]. The health center does not have enough supply, either*.”.

(senior citizen, 72 years old)

“*I know it is wrong to self-medicate, but home remedies are more natural and cheaper than my maintenance*.”.

(senior citizen, 66 years old)

##### Avoidance

Many senior citizens mentioned ignoring or avoiding their problems. They perceived avoidance tactics to be counterproductive when people resort to unhealthy habits, such as smoking, drinking, and gambling, to deal with stress.

“*I smoke and drink to free myself from stress. I know it is bad for my health. Also, I am not productive because I am not taking any actions to face my problems head-on*.”.

(senior citizen, 66 years old)

“*I play cards or bingo to forget all my worries. When I lost the game, it gave me more headache because I have no more money (laughs)*.”.

(senior citizen, 62 years old)

##### Homeboundness

A few senior citizens confined themselves within the comfort of their homes, typically due to illness or old age. They also preferred to stay at home so as not to bother their family members.

“*My sickness hinders me from going outdoors. I need assistance from my family members to help me move around. I do not want to bother them, so I prefer to stay at home. It is not safe to walk alone because I might fall*.”.

(senior citizen, 82 years old)

##### Longsuffering Attitude

Senior citizens were greatly influenced by spirituality, which allows them to endure suffering by leaving everything to God. All of them believed that this general optimism was a barrier in the assertion of their human rights, but this character trait has helped them endure their unmet needs.

“*I believe that those who endure trials are blessed because they will receive the crown of life which God has promised. So, I left everything to Him, and I am keeping my faith. I know he has better plans for me in the future*.”.

(senior citizen, 70 years old)

“*Although this character trait has helped us endure our unmet needs, it can hinder us from fighting for what we think we truly deserved*.”.

(senior citizen, 74 years old)

##### Overdependence

Some senior citizens relied on intergenerational family support. Their family members were providing their health care needs. Hence, they do not rely much on the government’s welfare services. However, a few of them have felt overdependence on their children’s financial support, and this situation might have triggered a sense of burden to their family. Such overdependence also negatively affects their self-esteem and dignity.

“*I do not want to be needy. I want to be needed by my family. However, sometimes, I feel like I am a burden because I always depend on them. They do not complain, but I can sense it. I have started losing my self-confidence*.”.

(senior citizen, 75 years old)

## 4. Discussion

Filipino senior citizens had a deep understanding of their unmet needs (Table 2). Four types of unmet needs emerged, which revolved around economic, health care, social services, and family support. Then, we explored their coping mechanisms (Table 3) to overcome these unmet needs. Senior citizens responded either positively or negatively to cope with the challenges associated with aging.

As shown in the results, senior citizens of this study had an unmet need for financial security, mainly due to the high rates of the informal workforce and the vicious cycle of poverty. They were not able to save for their pension as they had to consider their families’ expenses over their own needs. These results were in line with previous studies related to the labor market and social insurance coverage in the Philippines [30,31]. Ofreneo [30] reported that jobs in the large informal sector are increasingly precarious and generally unprotected, while Mandigma [31] mentioned poverty incidence as one of the drivers of pension coverage in the country. Hence, our results underscored the importance of extending social protection among informal workers and reducing poverty incidence to help achieve financial security in old age.

Despite positive economic growth in recent years, the levels of poverty, inequality, and vulnerability have remained stagnant in the Philippines [32]. As of 2018, the Philippine Statistics Authority reported a 21% poverty incidence or one out of five Filipinos is poor [33]. Also, senior citizens ranked sixth in the highest poverty rate among the eight basic sectors in the Philippines [34], and about 40 percent of them still do not have financial security [35]. This data shows that a vast number of Filipino senior citizens do not have the means necessary to sustain an adequate quality of life. Within this context, the government provides a social pension, but it only covered indigent senior citizens. Issues about the definition of ‘indigent’ and process of local validation transpired in the FGDs and in-depth interviews. Hence, to resolve the issues, Filipino senior citizens in this study suggested the provision of universal social pension. Other countries (such as Thailand, Vietnam, Timor-Leste, and Brunei) have provided one of the most effective and simplest ways of ensuring all senior citizens receive a pension regardless of the economic condition [36]. Evidence from these countries showed how universal social pension had huge impacts on poverty, livelihoods, and the economy as a whole [37,38]. However, senior citizens in this study expressed the low benefit level of social pension. Therefore, increasing the amount will give a stronger purchasing power to meet their basic needs, such as food and medicines.

Most of the senior citizens in this study rely on their families for their basic needs. However, the recent rapid economic and societal changes shaped the unmet need for family support. Senior citizens mentioned about OFW phenomenon, parenting by proxy, children’s lifelong dependency, and limited bonding time, as factors that influenced the reduction of family support. For Asian countries, family members are recognized as the primary source of care and financial support for senior citizens [39,40]. In the Philippines, for instance, the culture of ‘debt of gratitude’ strongly influences adult children’s sense of filial affection and moral obligation to their aging parents [41]. Thus, it is considered a grave social offense if children failed to support their parents when they get old [41,42,43]. However, our results revealed that the traditional support mechanism was slackening due to the socio-cultural changes experienced by Filipino families. This circumstance was especially true when children are too poor to support them. The worst part happened when children started to burden their parents, as in the case of ‘parenting by proxy’ and their ‘lifelong dependency’. This kind of family dynamics has become a trend in the Philippines nowadays and might add to the challenges faced by senior citizens.

Senior citizens of this study also expressed an unmet need for health care services and an age-friendly environment. Three factors hindered their access to health care services, including insufficient health workforce, inadequate supply of essential medicines, and a lack of accessible infrastructure. The Philippines has a ratio of one doctor for every 33,000 Filipinos [44], explaining why some neighboring barangays in this study were sharing their doctors. The lack of supplies at a health center can be explained by procurement inefficiencies and poor coordination between the Department of Health (DOH) and LGUs [45]. Hence, the present study calls for investing more in primary health care with the provision of doctors and medicines as vital components of the elderly program. Also, the accessibility of health centers was a concern among sick and frail senior citizens, implying the need to strengthen home care support services. On the other hand, senior citizens of this study expressed their need for safe housing conditions and social engagement to improve their quality of life. This result is supported by existing literature on the association between ‘perceived age-friendliness’ and quality of life among senior citizens [46].

To better understand how senior citizens address their unmet needs, it is crucial to look at their different coping strategies. Self-reliance and religious involvement were the two positive coping mechanisms practiced by Filipino senior citizens. They mentioned that being self-reliant boosts their self-esteem and gives them financial security. Many of them have a strong desire to continue working, but face barriers due to lack of opportunities. Our results indicated the need for employment opportunities for this particular age group to provide an additional social security safety net. Religious involvement, on the other hand, provided a source of joy and meaning to their lives. This result is in agreement with the literature that identifies the role of religion as a source of coping and resilience [47,48,49]. As for negative coping strategies, senior citizens used self-medication, avoidance, homeboundness, longsuffering attitude, and overdependence to cope with their unmet needs. They acknowledged that these could be counterproductive or have unintended negative consequences. For instance, self-medication helped them manage their illness, but its effects can be harmful and life-threatening. According to Ruiz [50], it is far from being a safe practice, especially in the case of non-responsible self-medication. On the other hand, being overly dependent on their family members for support might have triggered a sense of burden to their family. McPherson et al. [51] also reported this kind of ‘self-perceived burden’ among care recipients.

We acknowledged several limitations of this study. First, we conducted this study in one urban city located in the National Capital Region of the Philippines. Data collection from other subgroups located in the provinces will provide more information for the study. Second, due to the nature of this qualitative study, we might not generalize the results to more urban populations in the Philippines. Third, this study used convenience sampling, so there might be selection bias. BSNO staff and primary care physicians informed potential participants about the study so the results might be biased towards motivated participants. Fourth, although the FGD method provided us with rich data, we experienced challenges related to intimacy among senior citizens. To minimize the risk of participants being hidden in the crowd and dropping their inputs, we conducted the FGDs very carefully to get adequate responses from all of them. Finally, there is a risk of loss of information during the interviews since senior citizens might have had unmet needs other than those specified by our a priori theoretical framework. To overcome this issue, we treated our a priori themes as tentative, equally subject to redefinition or removal as any other theme.

## 5. Conclusions

This study is the first to explore the unmet needs and coping mechanisms of community-dwelling Filipino senior citizens from a qualitative perspective. Both focus group discussions and in-depth interviews from three local stakeholders gathered many profound insights about the unmet needs and coping mechanisms of senior citizens.

Currently, the Philippines is on the boundary of a demographic transition stage to an aging population. The country is not prepared to meet the needs of the influx of senior citizens. The government might consider revisiting existing national policies like the Senior Welfare Act to address their unmet needs. It is recommended to expand financial security, health care coverage, and age-friendly neighborhoods. Actions should be taken to strengthen positive coping and modifying the negative coping mechanisms, with a particular focus on community and family interventions.

## Figures and Tables

**Table 1:** Sampling details and research focus.

Type of Participants	Sampling Details	Age and Gender Profile	The Focus of FGDs or Interviews
Senior citizens	Four FGDs (*n* = 37) Group size: 7–10 members	Age range: 60–85 years More women participated (73% female, 27% male)	Demographic and socioeconomic status, physical and mental health status, health needs and health-seeking behavior, current economic and social security schemes, social participation and built environment, family relationships, coping strategies
Health providers (primary care physicians, nurses, midwives, BHWs)	Four FGDs (*n* = 15) Group size: 3–5 members	Age range: 38–69 years All participants were women	Organization profile, health and social services provided, health needs of the senior citizens, barriers in the implementation of programs
Local administrators (city mayor, OSCA representatives, city council staff, barangay captains)	Seven in-depth interviews (*n* = 7)	Age range: 38–65 years Only one administrator was a man	Organization profile, health and social services provided, health needs of the senior citizens, engagement of the senior citizens in community activities, barriers in the implementation of programs

Note: FGD—Focus Group Discussion; BHW—Barangay Health Worker; OSCA—Office of the Senior Citizens Affairs.

**Table 2:** An analytical framework of perception of unmet needs among Filipino senior citizens.

Theme	Categories	Description
Financial	Informal workforce	Never employed in any formal and regular jobs
security	Low household income	Prioritized food and other household needs over pension
	Lack of universal social pension	Social pension was only limited to indigent senior citizens
	Limited job opportunities	Would like to work but with limited job opportunities
Health care	Staffing problem	Lack of primary care physicians
services	Drug supply problem	Delay in the procurement of essential medicines
	Accessibility	Health center is far away from home
Age-friendly	Safety	Incidence of falls during the rainy season
environment	Poor housing condition	Lives in the squatter area
	Lack of social engagement	Suggested various daily activities at OSCA
Family	OFW phenomenon	Migration of traditional caregivers abroad
support	Parenting by proxy	Child-raising responsibilities imposed on aging parents
	Children’s lifelong dependency	Children had their own families but living with their parents
	Limited family bonding time	Reduction of face-to-face interaction in the family

Note: OFW—Overseas Filipino Worker.

**Table 3:** An analytical framework of coping mechanisms practiced by Filipino senior citizens.

Theme	Categories	Description
Positive	Self-reliance	Earned a small amount of money to satisfy basic needs
Coping	Religious involvement	Had a strong network of church friends
Negative	Self-medication	Used herbal supplements and decoctions to meet treatment demands
Coping	Avoidance	Resorted to unhealthy habits, such as smoking, drinking, and gambling
	Homeboundness	Preferred to stay at home so as not to bother their family members
	Longsuffering attitude	Endured suffering by leaving everything to God
	Overdependence	Depended too much on children’s financial support

## Supplementary Materials

The following are available online at https://www.mdpi.com/1660-4601/16/19/3745/s1, Table S1: Topic Guides (English); Table S2: Topic Guides (Filipino); Table S3: Consolidated criteria for reporting qualitative studies (COREQ): a 32-item checklist.
