# Supplementary material for: Unmet Needs and Coping Mechanisms Among Community-Dwelling Senior Citizens in the Philippines: A Qualitative Study
Source: Int J Environ Res Public Health. 2019 Oct 4;16(19):3745. doi: 10.3390/ijerph16193745 (PMC6801835; doi:10.3390/ijerph16193745)
Supplement: Supplementary file 1 [file ijerph-16-03745-s001.zip › Table S1. Topic Guides (English).pdf]

Table S1: Topic Guides (English)

A. Topic guides for senior citizens

Broad areas	Research Questions
Economic	1. What do you do for a living?<br>2. Are you receiving a monthly allowance scheme (e.g., pension)?<br>3. How do you feel about your economic situation?<br>4. What do you do to resolve your economic issues?
Health and Social Services	1. What do you think about your health status?<br>2. How do you feel the health care and social needs of older adults are being met in your community?<br>3. In general, what are the barriers, if any, to receiving or accessing the help you or others might need regarding your physical and mental health?<br>4. What do you do to address your health concerns?
Community	1. What can you say about your community?<br>2. What do you want from your community?<br>3. How does your community respond to your increasing needs and concerns?<br>4. What do you do for your community?
Environment (Physical and Social)	1. Tell us about your living arrangement.<br>2. Who are your main caregivers?<br>3. How age-friendly are the housing conditions?<br>4. Tell us about your participation in religious or social organizations.<br>5. What do you do for relaxation?
Household	1. How is your relationship with your family members?<br>2. How do they respond to your personal concerns or needs?<br>3. What do you do when your family members cannot respond to your personal concerns or needs?
General closing question	Is there any additional information that you would like to provide regarding the needs of the senior citizens in your community?

B. Topic guides for health providers and local administrators

Broad areas	Research Questions
Organization profile	1. What is the name of your organization and your role?<br>2. What is the mandate/focus of your organization?
Economic	Are the seniors covered by the monthly allowance scheme? Who might have been left out? If left out, reasons for it?
Health and Social Services	1. What services or programs does your agency provide for senior citizens?<br>2. Are there services in your city that are not accessible for senior citizens or are difficult to access? Why?<br>3. What programs or services for senior citizens do you think are missing in your city?<br>4. How can you provide comprehensive health care services to the senior citizens in the community? What types of coordination among external stakeholders can be possible?<br>5. What are the key challenges in providing comprehensive health care services the senior citizens in your community?
Community	Who do you think is the most vulnerable senior citizens in your community? Why are they in such a situation (determinants of the vulnerable situation)? How do senior citizens cope with the situation? Give an example.
Environment (Physical and Social)	1. What do you think about the housing conditions of the senior citizens living in your community?<br>2. How active are the senior citizens in your community?
Household	Do you think the role of the family as the caregiver of the senior citizens has been declining in your community? How and to what extent (youth migration, etc.)? Give an example.
General closing question	Is there any additional information that you would like to provide regarding the needs of the senior citizens in your community?
