# Supplementary material for: Unmet Needs and Coping Mechanisms Among Community-Dwelling Senior Citizens in the Philippines: A Qualitative Study
Source: Int J Environ Res Public Health. 2019 Oct 4;16(19):3745. doi: 10.3390/ijerph16193745 (PMC6801835; doi:10.3390/ijerph16193745)
Supplement: Supplementary file 1 [file ijerph-16-03745-s001.zip › Table S2. Topic Guides (Filipino).pdf]

Table S2: Topic Guides (Filipino)

<u>A. Topic guides para sa mga senior citizens</u>

Konsepto	Mga Katanungan
Economic	1. Ano ang inyong pinagkakabuhayan?<br>2. Kayo ba ay sakop ng buwanang *allowance* o benepisyong pinansiyal (halimbawa pensiyon)?<br>3. Ano sa palagay ninyo ang inyong kalagayang pinansiyal?<br>4. Anu-ano ang mga hakbang ang inyong ginagawa para matugunan ang inyong pangangailangang pinansiyal?
Pangkalusugan at Sosyal na Serbisyo	1. Ano ang masasabi ninyo sa inyong kalagayang pangkalusugan?<br>2. Sa tingin ba ninyo natutugunan ng inyong komunidad ang inyong mga pangangailangang pangkalusugan?<br>3. Sa kabuuan, ano ang mga balakid bakit hindi magawa ng ilang *seniors* makakuha ng tulong para sa kanilang problemang pisikal at mental?<br>4. Anu-ano ang ang mga hakbang ang inyong ginagawa para matugunan ang inyong pangangailangang pangkalusugan?
Komunidad	1. Ano ang masasabi ninyo sa inyong komunidad?<br>2. Ano ang gusto ninyong makamit mula sa inyong komunidad?<br>3. Paano uma-aksiyon ang inyong komunidad upang matugunan ang inyong dumaraming *concern* at pangangailangan?<br>4. Anu-ano ang ang mga hakbang ang inyong ginagawa para sa inyong komunidad?
Kapaligiran (Pisikal at Sosyal)	1. Ilarawan ang inyong kondisyong pambahay.<br>2. Sino ang inyong pangunahing taga-pangalaga?<br>3. Gaano ka *age-friendly* ang inyong tirahan?<br>4. Ilarawan ang inyong partisipasyon sa mga *religious or social organizations*.<br>5. Anu-ano ang inyong ginagawa para ma-relaks?
Kabahayan	1. Ilarawan ang inyong relasyon sa iyong mga kasambahay.<br>2. Paano nila ginagampanan ang inyong mga pangangailangan?<br>3. Anu-ano ang ang mga hakbang ang inyong ginagawa kung sakaling hindi matugunan ng inyong mga kasambahay ang inyong mga *concern* at pangangailangan?
Pangwakas na katanungan	Mayroon pa bang karagdagang impormasyon na gusto ninyong ilahad tungkol sa mga pangangailangan ng mga *seniors* sa inyong komunidad?

B. Topic guides para sa mga health providers at local administrators

Konsepto	Mga Katanungan
Profile ng Organisasyon	1. Anong pangalan ng inyong organisasyon at ano ang iyong responsibilidad?<br>2. Ano ang layunin ng inyong organisasyon?
Economic	Ang mga *seniors* ba ay sakop ng buwanang *allowance* o benepisyong pinansiyal? Sino ang mga hindi kabilang? Ano ang mga posibleng dahilan kung bakit hindi nagawang mapabilang?
Pangkalusugan at Sosyal na Serbisyo	1. Anong mga serbisyo o programa ang ginagawa ng inyong ahensiya para sa mga *seniors*?<br>2. Mayroon bang mga serbisyo sa lungsod na hindi aksesibol o mahirap maaccess ng mga *seniors*? Bakit?<br>3. Anong programa o serbisyo para sa mga *seniors* ang sa tingin mo ay wala sa lungsod?<br>4. Paano tayo makakapagbigay ng komprehensibong pangangalaga sa mga *seniors* sa ating komunidad? Anong klaseng hakbang ang dapat gawin para maisakatuparan ito?<br>5. Ano ang pangunahing mga balakid para makamit ang komprehensibong pangangalaga sa mga *seniors* sa inyong nasasakupan?
Komunidad	Sino sa tingin ninyo ang pinakavulnerable na *seniors* sa inyong lugar? Bakit sila nasa ganiyang kalagayan? Paano nila nakakaya ang ganiyang sitwasyon? Magbigay ng halimbawa
Kapaligiran (Pisikal at Sosyal)	1. Ano ang inyong masasabi hinggil sa kalagayang pambahay ng mga *seniors* sa inyong komunidad?<br>2. Gaano ka-aktibo ang mga *seniors* sa inyong komunidad?
Kabahayan	Sa tingin ninyo ang responsibilidad ba ng pamilya para alagaan ang mga *seniors* ay unti-unti nang humihina? Sa paanong paraan na isasantabi ang pangangalaga sa mga *seniors*? Magbigay ng halimbawa.
Pangwakas na katanungan	Mayroon pa bang karagdagang impormasyon na gusto ninyong ilahad tungkol sa mga pangangailangan ng mga *seniors* sa inyong komunidad?
