# Supplementary material for: Unmet Needs and Coping Mechanisms Among Community-Dwelling Senior Citizens in the Philippines: A Qualitative Study
Source: Int J Environ Res Public Health. 2019 Oct 4;16(19):3745. doi: 10.3390/ijerph16193745 (PMC6801835; doi:10.3390/ijerph16193745)
Supplement: Supplementary file 1 [file ijerph-16-03745-s001.zip › Table S3. COREQ.pdf]

Table S3: Consolidated criteria for reporting qualitative studies (COREQ): a 32-item checklist

Title： Unmet needs and coping mechanisms among community-dwelling senior citizens in the Philippines: a qualitative study

Authors: Rogie Royce Carandang, Edward Asis, Akira Shibanuma, Junko Kiriya, Hiroshi Murayama, Masamine Jimba

No. Item	Guide questions/description	Response
**Domain 1: Research team and reflexivity**		
Personal Characteristics		
1. Interviewer/facilitator	Which author/s conducted the interview or focus group?	Rogie Royce Carandang (RRC) and Edward Asis (EA)
2. Credentials	What were the researcher's credentials? E.g., Ph.D., MD	RRC: RPh, MPH, MSc, PhD<br>EA: MA<br>AS: MID, PhD, Assistant Professor<br>JK: PhD., Assistant Professor<br>HM: RN, PHN, PhD, Project Lecturer<br>MJ: MD, MPH, PhD, Professor, and Chair
3. Occupation	What was their occupation at the time of the study?	Researcher and faculty members
4. Gender	Was the researcher male or female?	Five males and one female
5. Experience and training	What experience or training did the researcher have?	RRC and EA have done previous several qualitative research projects.<br>JK, AS, and MJ have current community-based research projects. HM has aging research projects.
Relationship with participants		
6. Relationship established	Was a relationship established before study commencement?	JK, AS, HM, and MJ had no relationships with the participants before the study, while RRC and EA knew the community leaders through another community-based research.
7. Participant knowledge of the interviewer	What did the participants know about the researcher? e.g., personal goals, reasons for doing the research	Participants knew about the two interviewers' names and affiliation.
8. Interviewer characteristics	What characteristics were reported about the interviewer/facilitator? e.g., bias, assumptions, reasons, and interests in the research topic	Participants did not know about interviewers' characteristics except their names and affiliation.

Domain 2: Study design		
Theoretical framework		
9. Methodological orientation and Theory	What methodological orientation was stated to underpin the study? e.g., grounded theory, discourse analysis, ethnography, phenomenology, content analysis	See the manuscript in the method section/study design.
Participant selection		
10. Sampling	How were participants selected? e.g., purposive, convenience, consecutive, snowball	See the manuscript in the method section/participants.
11. Method of approach	How were participants approached? e.g., face-to-face, telephone, mail, email	See the manuscript in the method section/participants.
12. Sample size	How many participants were in the study?	59 participants.<br>See the manuscript in the method section/data collection and results section.
13. Non-participation	How many people refused to participate or dropped out? Reasons?	No one has dropped out.
Setting		
14. The setting of data collection	Where was the data collected? e.g., home, clinic, workplace	For FGDs and in-depth interviews, four communities in one urban city in the National Capital Region.<br>See the manuscript in the method section/data collection.
15. Presence of nonparticipants	Was anyone else present besides the participants and researchers?	Yes. One senior female volunteer
16. Description of sample	What are the important characteristics of the sample? e.g., demographic data, date	See the manuscript in the method section/participant and focus group and results.
Data collection		
17. Interview guide	Were questions, prompts, guides provided by the authors? Was it pilot tested?	There was no pilot testing.<br>See the manuscript in the method section/ data collection/semi-structured interview guide.
18. Repeat interviews	Were repeat interviews carried out? If yes, how many?	No.
19. Audio/visual recording	Did the research use audio or visual recording to collect the data?	Data were audio-recorded.<br>See the manuscript in the method section/ data collection.
20. Fieldnotes	Were field notes made during and/or after the	Yes, field notes were made during interviews and FGDs.

	interview or focus group?	
21. Duration	What was the duration of the interviews or focus group?	Approximately 60–90 minutes. See the manuscript in the method section/ data collection.
22. Data saturation	Was data saturation discussed?	Yes. See the manuscript in the method section/data collection.
23. Transcripts returned	Were transcripts returned to participants for comment and/or correction?	No.

**Domain 3: Analysis and findings**		
Data analysis		
24. Number of data coders	How many data coders coded the data?	Two, RRC and EA.
25. Description of the coding tree	Did authors provide a description of the coding tree?	No.
26. Derivation of themes	Were themes identified in advance or derived from the data?	See the manuscript in the method section/ data analysis.
27. Software	What software, if applicable, was used to manage the data?	NVivo 10® software was used to code and manage the data.
28. Participant checking	Did participants provide feedback on the findings?	Yes. See the manuscript in the method section/ data analysis.
Reporting		
29. Quotations presented	Were participant quotations presented to illustrate the themes/findings? Was each quotation identified? e.g., participant number	Yes. See the manuscript in the results.
30. Data and findings consistent	Was there consistency between the data presented and the findings?	Yes. See the manuscript in the data analysis/the trustworthiness of the study.
31. Clarity of major themes	Were major themes clearly presented in the findings?	Yes. See the manuscript in the results.
32. Clarity of minor themes	Is there a description of diverse cases or a discussion on minor themes?	Yes. See the manuscript in the results.

1. Developed from: Tong, A.; Sainsbury, P.; Craig, J. Consolidated criteria for reporting qualitative research (COREQ): a 32-item checklist for interviews and focus groups. *Int J Qual Health Care* **2007**, *19*, 349-357.
